# Primary glioblastoma of the cauda equina with molecular and histopathological characterization: Case report

**DOI:** 10.1093/noajnl/vdab154

**Published:** 2021-10-19

**Authors:** Rebekah G Langston, Christopher P Wardell, Angela Palmer, Hayden Scott, Murat Gokden, T Glenn Pait, Analiz Rodriguez

**Affiliations:** 1 Department of Neurosurgery, University of Arkansas for Medical Sciences, Little Rock, Arkansas, USA; 2 Department of Biomedical Informatics, University of Arkansas for Medical Sciences, Little Rock, Arkansas, USA; 3 Division of Neuropathology, Department of Pathology, University of Arkansas for Medical Sciences, Little Rock, Arkansas, USA

**Keywords:** cauda equina, glioblastoma, nerve root entry zone

*IDH*-wildtype glioblastoma (GBM) is an aggressive brain tumor of astrocytic origin that remains incurable. With prognosis of GBM in any location improving little over the last 40 years,^1^ extensive efforts are being made to identify novel therapeutic targets through molecular characterization of these neoplasms. Gene networks implicated in GBM pathogenesis commonly include growth factor signaling (PI3K) and tumor suppressor (p53, Rb) pathways as well as those involved in telomere regulation.^2–4^ Although GBM is the most common malignant primary central nervous system (CNS) tumor in adults,^5^ incidence of primary lesions below the tentorium is low. In a study of more than 23 000 GBM patients, the tumor was located in the cerebellum in only 0.9% of cases.^6^ Spinal GBM is even more rare with an approximate incidence of 0.1%, as spinal cord tumors account for only 5%-8% of all CNS tumors and only 1.5%-1.9% of all spinal cord tumors are classified as GBM.^7,8^ Occurrence of primary GBM at a nerve root is exceedingly rare with only a few histologically defined cases reported in the literature to date: three associated with cranial nerve (CN) XIII,^9–11^ one with CN III,^12^ and one with CN V^13^ (Table 1). Here, we describe the case of a primary GBM lesion originating in the cauda equina in a 76-year-old female known to have invasive breast cancer. We report both the histopathological and molecular features of this unique tumor, in the first report of a primary cauda equina GBM to our knowledge.

**Table 1.** Reported Cases of Primary GBM Lesions Originating From CNS Nerve Structures

Authors and Year	Diagnosis	Associated Nerve	Age (yr)	Sex	Treatment	Postoperative Survival	Molecular Findings
Wu et al., 2011	GBM	CN XIII	60	M	Subtotal resection	2 mo	Histopathology only
Yang et al., 2018	GBM	CN XIII	55	M	Subtotal resection	2.5 mo	Histopathology only
Takami et al., 2018	GBM	CN XIII	55	M	Near-total resection, CTX, RT	>5 mo	*TERT* (C250T), *TP53* (c.659A>G), *NF1* (c.480-1G>A), *RB1* (c.951_954del), *PIK3R1* (c.1126G>A); complex molecular karyotype
Reifenberger et al., 1996	GBM	CN III	70	F	Subtotal resection, RT	6 wk	Histopathology only
Breshears et al., 2015	GBM	CN V	67	M	Biopsy, CTX, RT	>23 wk	Histopathology only
Present study	GBM	Cauda equina	76	F	Subtotal resection	1 mo	*TERT* (c.-124C>T), *PIK3R1* (p.L380fs), CDK4 amp, MDM2 amp

Abbreviations: CN, cranial nerve; CNS, central nervous system; CTX, chemotherapy; GBM, glioblastoma; RT, radiation

## Case Report

**Figure 1.**
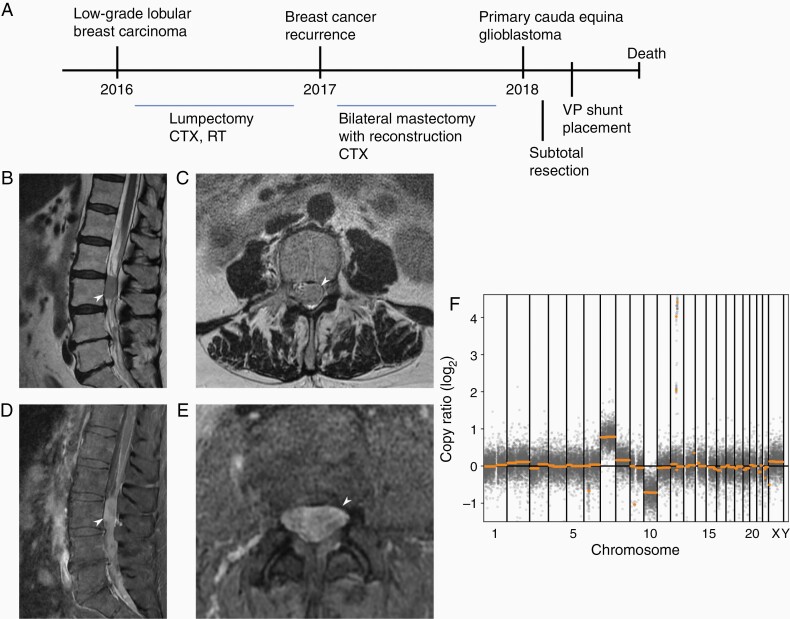
(A) Timeline of patient disease progression and treatment interventions. Sagittal (B) and axial (C) T2-weighted images, and sagittal (D) and axial (E) contrast-enhanced T1-weighted images of the lumbosacral spine were obtained on patient presentation in 2018. The 1.2 × 1.3 × 2.3 cm enhancing intradural extramedullary mass posterior to L3 is indicated by white arrows. (F) Copy number variation plot demonstrating chromosomal derangements present in the lesion.

A 76-year-old female presented with intractable right-sided low back pain that radiated to her right leg in 2018. She had a history of locally invasive low-grade lobular carcinoma of the left breast, diagnosed in 2016. It was found to be ER+, PR+, and HER2/neu+ with a Ki-67 of 3%, and was treated with lumpectomy followed by chemotherapy (Taxol and Herceptin) and radiation. She was subsequently found to have recurrent disease in 2017 and underwent bilateral mastectomy with reconstruction. She had been on daily chemotherapy (Anastrozole) since that time. A timeline illustrating this patient’s disease course is shown in Figure 1A. On presentation in 2018, imaging of the lumbar spine was obtained that showed extensive leptomeningeal enhancement throughout the entire spine along with a 1.2 × 1.3 × 2.3 cm enhancing intradural mass posterior to L3 which was displacing the adjacent nerve roots (conus medullaris terminated at T12-L1; Figure 1B–E). There was additional “sugar-coating” metastatic involvement of the distal spinal cord, but no abnormal enhancement in the brain. Initially, this was thought to be leptomeningeal spread of her breast cancer. The patient underwent L2-L3 laminectomies with resection of the intradural tumor at L3; the pathology was consistent with GBM. A month later, the patient experienced fluctuations in mental status, and imaging demonstrated hydrocephalus for which she underwent emergent right parietal ventriculoperitoneal shunt placement. MRI at that time confirmed the absence of intracranial obstructive lesions indicating that the etiology of the non-obstructive hydrocephalus may be related to the development of reactive arachnoiditis, increased CSF protein levels, and/or undetected leptomeningeal extension of the cauda equina lesion. She improved briefly, but then experienced a rapid decline in mental status despite multiple shunt revisions. The patient was discharged to hospice care and subsequently expired in December of 2018. An autopsy was performed further confirming no evidence of GBM within the brain or spine parenchyma with only involvement in the cauda equina.

Molecular characterization was completed using the Tempus xT oncology next-generation sequencing panel with a median sequencing depth of 1847X across all 596 target regions. The tumor was *IDH* wild-type. Whole chromosome events within the lesion included loss of chromosome 10 and gain of chromosome 7 (Figure 1F). Amplification of 2 genes on chromosome 12, *CDK4* and *MDM2*, was also observed. The largest copy number gains and losses are further described in Supplementary Table 1, including the aforementioned events as well as copy number losses of unknown significance on chromosomes 6 and 9. In addition to these pathologic copy number variations, 2 mutations likely contributing to tumorigenesis were identified: (1) a loss-of-function frameshift mutation in *PIK3R1* (p.L380fs) with a variant allele fraction (VAF) of 31.6% and (2) a promoter mutation in *TERT* (c.-124C>T) with VAF of 28.6%. A total of 29 single-nucleotide variants (SNVs) and 10 insertion-deletion (indel) mutations were found, with 2 SNVs and 3 indels predicted to have deleterious consequences (Supplementary Table 2). Of note, *PIK3R1* is potentially biallelically inactivated with tumor cells sustaining both a frameshift indel and a rare missense SNV. Altogether, the cauda equina GBM lesion was found to have genetic perturbations in multiple pathways implicated in GBM development,^2,4^ including the Rb pathway (*CDK4*), the PI3K pathway (*PIK3R1*), the p53 pathway (*MDM2*), and telomerase activation (*TERT*).

**Figure 2.**
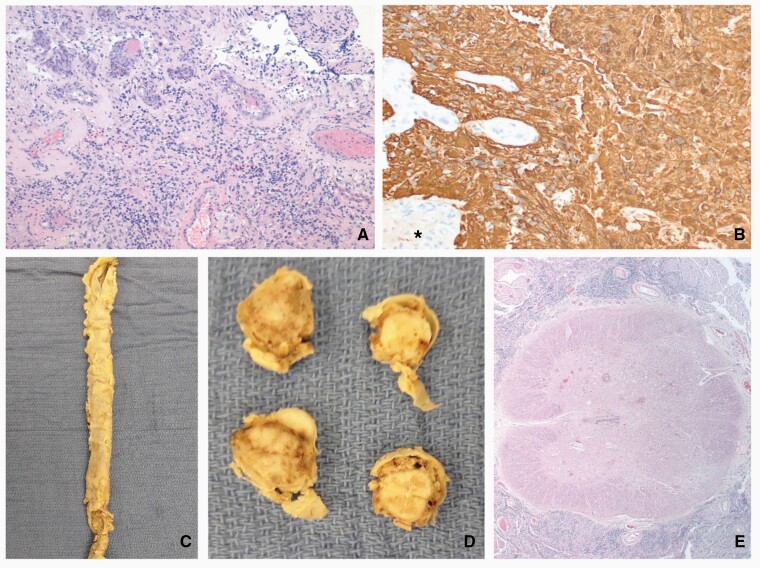
Pathologic findings: the glial neoplasm (A) identified in the resection specimen was *GFAP*-positive (B) and had vascular proliferation (*). Spinal cord from the autopsy showed the spinal dura mater to be expanded by the subarachnoid neoplasm (C) that surrounded the spinal cord but did not infiltrate it on cross-sections grossly (D) or microscopically (E). Original magnifications: A, 100×; B, 200×; E, 20×.

Histopathological examination of the resected L3 intradural, extramedullary lesion showed a glial neoplasm with areas of necrosis and vascular proliferation. The neoplasm was diffusely positive for *GFAP* upon immunohistochemical analysis, with a Ki-67 of 30%-40% (Figure 2A and B). Although the neoplasm encircled and infiltrated the spinal nerve roots, there was no parenchymal infiltration. The differences in the histologic features and the *GFAP*-positive, cytokeratin-negative nature of the spinal neoplasm ruled out a metastasis from the patient’s previously diagnosed breast carcinoma. Notably, neoplastic cells were also *GATA3*-negative and therefore not suggestive of metastatic breast carcinoma. The diagnosis determined by histopathology was thus intradural extramedullary GBM, WHO grade IV. Subsequent autopsy showed the spinal subarachnoid space to be engorged by abundant glial neoplasm (Figure 2C–E). It was more prominent in the lumbosacral region than in the thoracic and cervical levels. It was again seen to entrap and infiltrate the spinal nerve roots. A rare superficial infiltration of the neoplasm along the Virchow-Robin spaces was identified, but no parenchymal invasion was present. No spinal or intracranial parenchymal mass lesions or gliomatosis pattern of involvement was found.

## Discussion

Here, we report a case of primary GBM originating in the cauda equina in a female patient with a history of invasive breast carcinoma. Primary neoplasms involving the cauda equina are most commonly schwannomas and ependymomas.^14^ Although 1.7% of primary cauda equina tumors (5/293) reported to the National Cancer Institute’s Surveillance, Epidemiology, and End Results (SEER) database were categorized as GBM,^14^ no other reports of cauda equina GBM cases were found upon review of the literature highlighting the unique nature of a tumor occurring in this location. GBM lesions are usually supratentorial, occurring most frequently in the frontal lobe or across multiple cerebral lobes.^15^ GBM of the cerebellum is relatively rare and GBM of the spinal cord is very rare,^1^ and there have been only a few reports of nerve root GBM.^9–13^ The nerve root entry zone of both cranial and spinal nerves is known to contain glial tissue,^16^ allowing for the possibility of primary gliomas arising from nerves. Indeed, primary CN root gliomas have been recognized as a rare subset of cerebellopontine angle tumors.^17^

In the present case of a GBM apparently arising from the nerve roots comprising the cauda equina, consideration of alternate origins of the tumor is warranted. Development via subarachnoid spread of a primary lesion located elsewhere in the CNS is unlikely as no parenchymal lesions were detected in the brain or spine, and the cauda equina GBM was confirmed to be extramedullary. Additionally, the lesion is not likely to have originated through a process related to the patient’s breast cancer diagnosis or treatment as it strongly and diffusely expressed *GFAP*, it did not express *GATA3* (a marker typically detected in breast cancer metastases), and its location was outside of the field of the patient’s previous radiation treatment. We therefore conclude that the likely site of origin for this GBM is the spinal nerve root entry zone, also known as the Obersteiner-Redlich zone, in the region of transition between the spinal cord parenchyma enveloped by glial cells, and the peripheral nerves covered by Schwann cells. Given the unusual occurrence of primary GBM in the cauda equina, we look to molecular characterization of the tumor for further insight.

The L3 intradural extramedullary lesion was consistent with GBM at both histopathological and molecular levels. A study of *IDH* wild-type GBM tumorigenesis found that gain of chromosome 7, loss of 9p, or loss of 10 was commonly found in the early phase of tumor growth, with *TERT* promoter mutations later initiating a rapid growth phase.^3^ In the present case, gain of chromosome 7, loss of chromosome 10, as well as an SNV in the *TERT* promoter region were detected. Activation of telomerase, encoded by the *TERT* gene, is a key step in oncogenesis as persistent maintenance of telomere length prevents normal cell aging and promotes cell immortality.^18^ The c.-124C>T variant is a “hotspot” mutation of the *TERT* promoter documented in multiple cancer types, including GBM, that results in increased transcription of the gene.^19,20^*TERT* promoter mutations have been associated with poor prognosis in *IDH* wild-type GBM.^21^ The described cauda equina lesion thus contains several genetic features expected to facilitate GBM growth.

Additional disturbances were identified in genes associated with 3 major pathways known to be involved in GBM pathogenesis. A loss-of-function mutation was seen in *PIK3R1* (p.L380fs) which encodes the p85α regulatory subunit of phosphoinositide 3-kinase (PI3K). About 25% (63/251) GBM contained PI3K mutations in a report of somatic alterations commonly observed in GBM generated by The Cancer Genome Atlas Research Network (TCGA).^2^ PI3K is, like telomerase, often involved in oncogenesis with increased PI3K signaling being a classic feature of multiple cancers,^22^ leading to the evaluation of PI3K pathway inhibitors as potential novel cancer treatments. Next, amplifications of both the *CDK4* gene and the *MDM2* gene were observed on chromosome 12, indicating dysregulation of the Rb and p53 tumor suppressor pathways, respectively. These copy number gains contribute to uncontrolled cell proliferation, and are commonly observed events in GBM lesions with *MDM1/2/4* amplification found in 15.1% and *CDK4/6* amplification found in 15.5% of the TCGA GBM patient cohort.^2^ The molecular findings in the primary cauda equina GBM are consistent with those observed in previous studies, and add to our understanding of the complex mechanisms leading to GBM tumorigenesis.

## Supplementary Material

vdab154_suppl_Supplementary_Tables
