# Supplementary material for: Primary glioblastoma of the cauda equina with molecular and histopathological characterization: Case report
Source: Neurooncol Adv. 2021 Oct 19;3(1):vdab154. doi: 10.1093/noajnl/vdab154 (PMC8577522; doi:10.1093/noajnl/vdab154)
Supplement: vdab154_suppl_Supplementary_Tables [file vdab154_suppl_supplementary_tables.docx]

**Supplementary Tables**

| Genomic coordinates (GRCh37) | Size (Mb) | Loss or gain | Candidate genes in region |
| --- | --- | --- | --- |
| 6:44051502-54354271 | 10.3 | Loss |  |
| 7 | 159.1 | Gain | Whole chromosome |
| 9:39079042-46893601 | 7.8 | Loss |  |
| 10 | 135.5 | Loss | Whole chromosome |
| 12:58142301-58893019 | 0.75 | Gain | CDK4 |
| 12:69052346-70270150 | 1.2 | Gain | MDM2 |

Supplementary Table 1. Copy number aberrations larger than 0.5 megabases (Mb) and with an absolute copy ratio of greater than 0.5.

| Gene | Genomic coordinates | HGVS DNA Reference | HGVS Protein Reference | Variant Type | Predicted Effect | dbSNP ID | Genotype |
| --- | --- | --- | --- | --- | --- | --- | --- |
| PIK3R1 | 5:67589147 | ENST00000521381.1:c.1139_1142del | ENSP00000428056.1:p.Leu380SerfsTer16 | Indel | Frameshift |  | Heterozygote |
| PIK3R1 | 5: 67590463 | ENST00000521381.1:c.1525A>G | ENSP00000428056.1:p.Ile509Val | SNV | Missense | rs763729975 | Heterozygote |
| WRN | 8:31004874 | ENST00000298139.5:c.3460-6_3463delinsTTTTTTTTAT | ENSP00000298139: p.IleVal1154LeuLeu | Indel | Splice acceptor |  | Heterozygote |
| DYNC2H1 | 11:102987295 | ENST00000398093.3:c.622-4_624delinsTGTTTTT | ENSP00000381167: p.Glu208Phe | Indel | Missense |  | Heterozygote |
| BRCA1 | 17: 41234422 | ENST00000471181.2:c.4356A>T | ENSP00000418960.2:p.Lys1452Asn | SNV | Missense |  | Heterozygote |

Supplementary Table 2. All variants with non-synonymous consequences that were detected in this case. Gene coordinates given in GRCh37 genome build.
